# Isolated Bilateral Ptosis as an Early Sign of Guillain-Barré Syndrome

**DOI:** 10.1155/2013/178291

**Published:** 2013-03-25

**Authors:** Yahia Z. Imam, Dirk Deleu

**Affiliations:** Department of Neurology (Medicine), Hamad Medical Corporation, P.O. Box 3050, Doha, Qatar

## Abstract

*Background*. Guillain-Barré syndrome (GBS) has many variants with distinct presentations. Ptosis as an initial presentation is rare. *Case Report*. We describe a young female with bilateral ptosis without ophthalmoplegia as the initial presentation of Guillain-Barré ptosis in an anti-GQ1b IgG antibody negative patient with a favorable outcome to intravenous immunoglobulins. *Objectives*. Our paper highlights the importance of recognizing GBS as a potential etiology in a patient presenting with isolated ptosis, particularly since the course of GBS can be more dramatic than in the anti-GBQ1b syndromes such as ophthalmoparesis without ataxia and Miller Fisher syndrome or ocular myasthenia. *Conclusion*. This is the first paper of anti-GBQ1b antibody negative GBS presenting with isolated ptosis without ophthalmoparesis. GBS should be included in the list of differential diagnosis of such presentations.

## 1. Introduction

Guillain-Barré syndrome (GBS) is an acute inflammatory polyradiculoneuropathy characterized by rapidly developing motor weakness. It is now recognized as an increasingly heterogeneous disorder with several variants, each with its distinctive features. The most common form is the acute inflammatory demyelinating polyneuropathy (AIDP), while other less common variants are the axonal subtypes, that is, acute motor axonal neuropathy (AMAN), acute motor and sensory axonal neuropathy (AMSAN), and Miller Fisher syndrome [1].

 Over half of the patients with GBS may develop cranial nerve involvement, commonly facial weakness. Ocular involvement and in particular isolated ptosis without ophthalmoplegia are a rare manifestation. 

We report a young female presenting with isolated bilateral ptosis as an initial manifestation of GBS. To our knowledge, bilateral ptosis as an early manifestation of a GBS is rare and may pose considerable diagnostic challenges.

## 2. Case Report

A 30-year-old housewife presented with a one-week history of progressive painless bilateral ptosis. Initially the ptosis affected the left eye and was followed five days later by ptosis of the right eye. She also noticed gradual-onset malaise and fatigability upon doing her regular activities progressing to decreasing dexterity in both upper limbs and fatigability on walking. She denied any preceding history of flu-like symptoms or gastrointestinal infection and no diurnal fluctuation in her symptoms were present. Her medical history was otherwise unremarkable.

### 2.1. Vital Signs Were within Normal Limits

**Figure 1:**
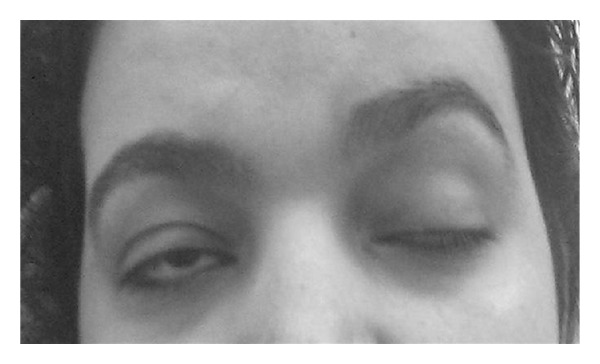
At first presentation, bilateral ptosis more pronounced on the left.

Neurological examination revealed normal mental status. There was bilateral ptosis (Figure 1) with inability to open the lids volitionally. Upward gaze produced mild lid elevation. The pupils were equal and reactive to light, and there was no restriction in horizontal and vertical eye movement, and Bell's phenomenon was preserved. Neurological examination of the cranial nerves was otherwise normal. Sensory examination was normal. Muscle strength, tone, coordination, and gait were normal. Deep tendon reflexes were absent. 

Complete blood cell count and biochemical laboratory screening (renal, liver and thyroid function, electrolyte, and glucose) were normal. Since ocular myasthenia gravis needed to be ruled out, an edrophonium test was performed but was negative, and repetitive supramaximal stimulation (SRS) testing on day 9 of the illness with recording from the right abductor digiti minimi (ADM) and the both biceps did not show a decremental response. Cerebrospinal fluid (CSF) analysis yielded albuminocytological dissociation with a protein of 0.6 gm/L (normal 0.15–0.45 gm/L) and less than 5 white blood cells/uL (normal < 5/ul). CSF glucose was normal. CSF viral serology and gram stain and culture as well as tuberculosis smear and culture were negative. Serum anti-GQ1B, antiganglioside and antiacetylcholine receptor antibodies sent on day 12 of the illness (before initiating treatment) were negative. 

Magnetic resonance imaging (MRI) of the brain was normal. Nerve conduction study (NCS) (Tables 1 and 2) performed on day 10 of the illness revealed significant reduction in the compound motor action potentials (CMAP) with conduction blocks in the median and ulnar nerves and reduced motor conduction velocity (MCV) in the lower limbs with reduced CMAP amplitudes. The *F* wave was preserved but dispersed in the upper limbs and absent in the lower limbs. Electromyography (EMG) showed diffuse signs of denervation in all myotomes of the face, upper limbs, and lower limbs. In addition, significant polyphasia was noted in the muscles of the face. These findings were consistent with acute predominantly axonal inflammatory polyradiculoneuropathy.

**Figure 2:**
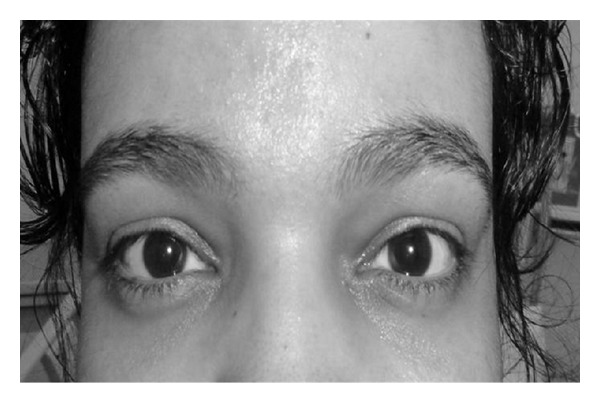
Resolution of ptosis after treatment with IVIG.

 By that time (day 10 of the illness), the patient started developing a gradual increase in lower limb weakness and had increasing difficulty in walking. The patient was started on intravenous immunoglobulin (IVIG) therapy (0.4 g/kg/day IV for 5 days) with rapid and complete resolution of her neurological findings (Figure 2). 

## 3. Discussion

In this patient, there were several findings which supported the diagnosis of a variant of GBS over Miller Fisher syndrome: firstly, the presence of albuminocytological dissociation in the first week, which is unusual for Miller Fisher syndrome [2]; secondly, the absence of anti-GQ1b IgG antibodies; and finally, the clinical course, namely, the absence of opthalmoplegia throughout the course.

Acute ptosis can be a diagnostic challenge. From a neurological perspective, the etiology of bilateral ptosis can range from central causes secondary to right hemispheric pathology [3], lesions in midbrain affecting the oculomotor complex, lesions of the oculosympathetic pathway, and lesions in neuromuscular junction as in myasthenia and botulism. All these causes were excluded by neuroimaging or electrodiagnostic testing. Furthermore, the patient did not fulfill the criteria for Miller Fisher syndrome, GBS with ophthalmoplegia, Bickerstaff's brain stem encephalitis or acute ophthalmoparesis without ataxia (AO) [4]. The preservation of autonomic pupillary function excludes botulism intoxication.

Acute isolated bilateral ptosis without ophthalmoplegia is more commonly observed in ocular myasthenia gravis. In AO, one of the so-called anti-GQ1b IgG antibody syndromes, the most common manifestation is external ophthalmoplegia (bilateral abduction deficit), followed by oculomotor nerve involvement, internal ophthalmoplegia, and finally ptosis [5] which is not the case here.

 Odaka et al. reported ptosis in less than 45% of AO patients [4]. All of these patients had associated symptoms of external ophthalmoplegia. A single report describes a pediatric case of isolated ptosis in AO associated with anti-GQ1b IgG antibodies [6]. 

In our case, GBS presenting as an isolated ptosis without ophthalmoplegia in anti-GQ1b IgG antibody negative patient has not been reported. Stalpers et al. reported a case of isolated bilateral ptosis and ataxia in a patient diagnosed with GBS [7]. No acute-phase anti-GQ1b IgG antibody sample was available. In their study, an anti-GQ1b IgG antibody sample taken 3 months after the symptom-onset and after having received intravenous immunoglobulin therapy was negative. Serum anti-GQ1b IgG antibodies have been shown to decline or disappear with clinical recovery [2, 8]. 

Ropper reported 8 patients with severe ptosis in GBS, three of these patients had this manifestation as an early sign of GBS, but all had associated external ophthalmoplegia and pupillary abnormalities [9]. Since this study was done in the era prior to anti-GQ1b IgG antibody testing, no data is available of the titers in these patients. 

Similarly, Teng and Sung [10] reported a case of ptosis as an early sign of “possible” GBS. No CSF analysis or serum anti-GQ1b IgG antibody testing was performed. In their case, the clinical presentation was more dramatic than ours prompting intubation and plasmapheresis.

Anti-GQ1B IgG antibodies are present in more than 85% of patients with Miller Fisher syndrome and GBS with ophthalmoplegia but are rarely found in GBS without ophthalmoplegia [4]. Furthermore, Lee et al. [5] observed isolated acute ophthalmoplegia in 32% of patients with anti-GQ1b IgG antibodies, ptosis presenting in 46% of them. 

The GQ1b ganglioside is a cell surface component that is concentrated in the paranodal regions of the human oculomotor, trochlear, and abducens nerves. It contains polysaccharides identical to the lipopolysaccharides contained in the outer membranes of certain bacteria and may be the target of an immune response initiated against epitopes shared by these nerve fibers [11].

Our paper highlights the importance of recognizing GBS as a potential etiology in a patient presenting with isolated ptosis, particularly since the course of GBS can be more dramatic than in the anti-GBQ1b syndromes such as AO and Miller Fisher syndrome or ocular myasthenia.

## 4. Conclusion

Isolated ptosis without ophthalmoparesis has a wide differential diagnosis. GBS should be included in the list. Several tests including anti-GBQ1b antibodies help narrow the differential diagnosis. This is the first paper of such presentation of GBS with negative anti-GBQ1b antibodies.

## Figures and Tables

**Table 1:** Nerve conduction study (motor).

Nerve	Latency (ms)		Amplitude (mV)		Conduction velocity (m/s)	
	Right	Left	Right	Left	Right	Left
Median						
Position 1	3.4	3.5	10.7	11.0		
Position 2	8.1	7.9	7.6	7.3		
Position 3	9.5	9.7	7.1	7.1	42.6	54.5
Position 4	14.2	—	0.8	—		
Ulnar						
Position 1	3.2	3.4	11.6	12.0		
Position 2	7.1	7.2	8.5	12.0		
Position 3	8.9	9.7	6.0	—	58.8	47.4
Position 4	10.6	—	4.9	—		
Tibial						
Position 1	5.7	5.6	1.8	3.4		
Position 2	17.4	15.0	1.2	2.0	29.9	38.3
Peroneal						
Position 1	5.3	5.1	3.0	4.7		
Position 2	14.2	13.8	3.1	3.5	36.0	37.9

**Table 2:** Nerve conduction study (sensory).

Nerve	Latency (ms)		Amplitude (micro V)		Conduction velocity (m/s)	
	Right	Left	Right	Left	Right	Left
Median	3.3	2.7	27	29	48.5	53.7
Ulnar	2.7	2.4	30	22	53.7	54.2
Sural	2.0	2.6	5.9	5.5	55.0	42.3

## References

[1] McGrogan A, Madle GC, Seaman HE, De Vries CS (2009). The epidemiology of Guillain-Barré syndrome worldwide: a systematic literature review. *Neuroepidemiology*.

[2] Nishimoto Y, Odaka M, Hirata K, Yuki N (2004). Usefulness of anti-GQ1b IgG antibody testing in Fisher syndrome compared with cerebrospinal fluid examination. *Journal of Neuroimmunology*.

[3] Averbuch-Heller L, Leigh RJ, Mermelstein V, Zagalsky L, Streifler JY (2002). Ptosis in patients with hemispheric strokes. *Neurology*.

[4] Odaka M, Yuki N, Hirata K (2001). Anti-GQ1b IgG antibody syndrome: clinical and immunological range. *Journal of Neurology Neurosurgery and Psychiatry*.

[5] Lee SH, Lim GH, Kim JS (2008). Acute ophthalmoplegia (without ataxia) associated with anti-GQ1b antibody. *Neurology*.

[6] Jindal G, Parmar VR, Gupta VK (2009). Isolated ptosis asacute ophthalmoplegia without ataxia, positive for Anti-GQ1b immunoglobulin G. *Pediatric Neurology*.

[7] Stalpers XL, Verhagen WIM, Meulstee J (2009). Isolated bilateral ptosis as the only ophthalmologic sign in the fisher variant of guillain-barré syndrome. *Journal of Neuro-Ophthalmology*.

[8] Yuki N, Sato S, Tsuji S, Ohsawa T, Miyatake T (1993). Frequent presence of anti-G(Q1b) antibody in Fisher’s syndrome. *Neurology*.

[9] Ropper AH (1986). Unusual clinical variants and signs in Guillain-Barre syndrome. *Archives of Neurology*.

[10] Teng HW, Sung JY (2012). Ptosis as the initial presentation of Guillain-Barré Syndrome. *The Journal of Emergency Medicine*.

[11] Chiba A, Kusunoki S, Obata H, Machinami R, Kanazawa I (1997). Ganglioside composition of the human cranial nerves, with special reference to pathophysiology of Miller Fisher syndrome. *Brain Research*.

